# Presence and Levels of Resilience and Its Sociodemographic Correlates in Older Chinese Americans

**DOI:** 10.1093/geroni/igaa057.1253

**Published:** 2020-12-16

**Authors:** Jieyang Zheng, Dexia Kong, Mengting Li, XinQi Dong

**Affiliations:** 1 Rutgers University, Chicago, Illinois, United States; 2 Rutgers University, New Brunswick, New Jersey, United States

## Abstract

Resilience is defined as a personal quality that enables growth in knowledge, understanding and self-actualization in the face of adversity and life disruptions. Low levels of resilience can dispose older adults to higher risks for negative health outcomes in the aftermath of traumatic events. However, we have limited knowledge of resilience among minority aging populations. This study aims to examine the presence and levels of resilience and its sociodemographic correlates among U.S. Chinese older adults. Data were drawn from the Population Study of Chinese Elderly, an epidemiology study of U.S. Chinese older adults in the Greater Chicago area. Guided by a community-based participatory research approach, a total of 3,036 Chinese older adults aged 60 and above participated in face-to-face interviews from 2015 to 2017. Spearman’s rank-order coefficient was utilized to test correlation. A 10-item validated Chinese version of the Connor-Davidson resilience scale was used to assess resilience. In our sample, 59.7% were female, and the average age was 75. The mean resilience score was 26.9, ranging from 1 to 40. U.S. Chinese older adults who were younger, male, married, had higher education and income, fewer children, better health status and quality of life, and improved health and have lived fewer years in the U.S. reported higher levels of resilience. Future longitudinal research is needed to investigate the protective effects of resilience among older Chinese Americans against mental and physical distress.

